# Genetically engineered pre-microRNA-34a prodrug suppresses orthotopic osteosarcoma xenograft tumor growth via the induction of apoptosis and cell cycle arrest

**DOI:** 10.1038/srep26611

**Published:** 2016-05-24

**Authors:** Yong Zhao, Mei-Juan Tu, Wei-Peng Wang, Jing-Xin Qiu, Ai-Xi Yu, Ai-Ming Yu

**Affiliations:** 1 Department of Orthopedics, Zhongnan Hospital of Wuhan University, Wuhan, Hubei, China; 2 Department of Biochemistry & Molecular Medicine, UC Davis School of Medicine, Sacramento, CA 95817, USA; 3 Department of Pathology, Roswell Park Cancer Institute, Buffalo, NY 14263, USA

## Abstract

Osteosarcoma (OS) is the most common primary malignant bone tumor in children, and microRNA-34a (miR-34a) replacement therapy represents a new treatment strategy. This study was to define the effectiveness and safety profiles of a novel bioengineered miR-34a prodrug in orthotopic OS xenograft tumor mouse model. Highly purified pre-miR-34a prodrug significantly inhibited the proliferation of human 143B and MG-63 cells in a dose dependent manner and to much greater degrees than controls, which was attributed to induction of apoptosis and G2 cell cycle arrest. Inhibition of OS cell growth and invasion were associated with release of high levels of mature miR-34a from pre-miR-34a prodrug and consequently reduction of protein levels of many miR-34a target genes including SIRT1, BCL2, c-MET, and CDK6. Furthermore, intravenous administration of *in vivo*-jetPEI formulated miR-34a prodrug significantly reduced OS tumor growth in orthotopic xenograft mouse models. In addition, mouse blood chemistry profiles indicated that therapeutic doses of bioengineered miR-34a prodrug were well tolerated in these animals. The results demonstrated that bioengineered miR-34a prodrug was effective to control OS tumor growth which involved the induction of apoptosis and cell cycle arrest, supporting the development of bioengineered RNAs as a novel class of large molecule therapeutic agents.

Osteosarcoma (OS) is the most common primary bone cancer consisting of malignant tumor cells that directly leads to osteoid or immature bone. OS accounts for approximately 60% of malignant bone tumors in children and young adults, and OS remains a leading cause of cancer-related death among adolescents^1^,^2^,^3^. OS is not only locally destructive but also highly pulmonary metastatic^4^,^5^,^6^. While there are some improved options including the combination of surgery and chemotherapy for the treatment of OS, 20–40% of OS patients are unable to survive over five years and most of them would eventually die of pulmonary metastases. Therefore, there is a clear need for developing more effective therapeutic strategies to treat OS. Nevertheless, current efforts are limited to the evaluation of small molecule drugs and protein therapeutics^7^.

MicroRNAs (miRNAs or miRs) are a large family of genomically encoded regulatory RNA molecules that may control various cancer cellular processes through the regulation of target gene expression^8^,^9^,^10^ and thus open the avenues to develop miRNA based therapy^10^,^11^,^12^. Recent studies have revealed that some miRNAs are dysregulated in OS tumor tissues and cancer cell lines, compared with normal bone tissues and osteoblastic cell lines, respectively^13^. Among them, miR-34a is significantly downregulated in OS tumors, and a lower miR-34a expression is also linked to a remarkably lower disease-free survival rate^14^,^15^,^16^. Indeed miR-34a acts as a tumor suppressor through the downregulation of many target genes in cancer cells, including NAD-dependent deacetylase sirtuin-1 (SIRT1), hepatocyte growth factor receptor c-MET, cyclin-dependent kinase 6 (CDK6), B-cell lymphoma 2 (BCL2), and cell surface glycoprotein CD44^17^,^18^,^19^,^20^,^21^. In addition, ectopic expression of miR-34a inhibits the proliferation and tumorigenesis of OS cells *in vitro* and *in vivo*, and reintroduction of chemically synthesized miR-34a mimics suppresses OS cell proliferation *in vitro*^15^,^16^,^22^,^23^. However, there is no report yet on the effectiveness of systemic administration of a miR-34a agent for the control of OS tumor progression in a whole body system.

In an effort to develop miRNA based therapy, we have established recently a novel approach to bioengineering of large quantities, more affordable pre-microRNA agents^24^,^25^,^26^,^27^,^28^. Different from synthetic miRNA agents (e.g., miR-34a mimics or pre-miR-34a)^11^,^16^,^19^,^29^,^30^, bioengineered RNA agents (BERAs) are produced and folded in the cells and may better capture the function and safety properties of natural RNAs and represent a novel class of large molecule agents for research and development^31^. Indeed genetically engineered miR-34a prodrug is selectively processed to mature miR-34a in human lung carcinoma cells, and consequently inhibits cancer cell proliferation *in vitro*. In addition, intratumoral injection of biological miR-34a prodrug sharply reduces the growth of subcutaneously implanted xenograft tumors *in vivo*^24^. In this study, we aimed to define the effectiveness and safety profiles of systemic administration of bioengineered miR-34a prodrug in a more clinically relevant orthotopic OS xenograft tumor mouse model *in vivo*. In addition, the mechanistic actions of biological miR-34a prodrug in the control of OS were delineated in human OS cells *in vitro*.

## Results

### Osteosarcoma cell proliferation is inhibited by genetically engineered miR-34a prodrug

**Figure 1:**
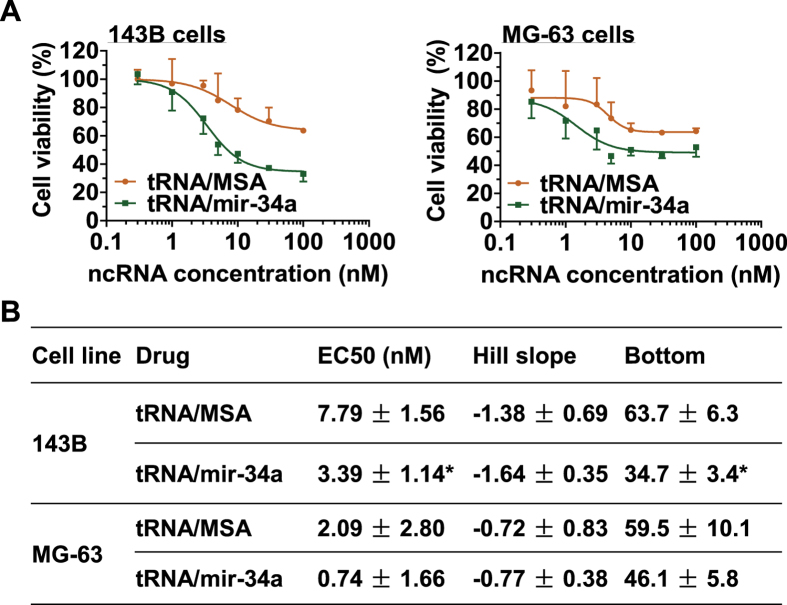
Inhibition of osteosarcoma cancer cell proliferation by genetically engineered miR-34a prodrug. (**A**) Dose-response curves for the effects of tRNA/miR-34a versus control tRNA/MSA on 143B and MG-63 cell viability. (**B**) The estimated pharmacodynamic parameters. Cell viability was determined using MTT assay at 72 h post-treatment, which was suppressed to a significantly (P < 0.001, two-way ANOVA) greater degree by tRNA/mir-34a than tRNA/MSA. This is also indicated by a lower EC50 and Bottom value (P < 0.05, one-way ANOVA). Values are mean ± SD of triplicate treatments.

We first employed MTT assay to assess the antiproliferative activity of miR-34a prodrug against human OS 143B and MG-63 cells. The results showed that tRNA/mir-34a suppressed osteosarcoma cell proliferation in a dose dependent manner, and to a significantly greater degree than the control tRNA/MSA (P < 0.001, two-way ANOVA; Fig. 1A). The greater effect of miR-34a prodrug on cell proliferation than the control tRNA/MSA was further manifested by lower EC50 and Bottom (greater inhibition) values, as compared with the control treatment (P < 0.05, one-way ANOVA; Fig. 1B). In addition, 143B cells were more sensitive to miR-34a prodrug than MG-63 cells (Fig. 1A), which was also indicated by the lower Bottom value (Fig. 1B). As such, and given the fact that the morphology of MG-63 cells is rather more fibroblastic and 143B cells are highly tumorigenic and metastatic^28^,^32^,^33^,^34^, the following *in vitro* mechanistic and *in vivo* therapeutic studies were conducted with 143B cells.

### BERA miR-34a prodrug is processed to mature miR-34a in OS cells and reduces the protein expression of many miR-34a target genes

**Figure 2:**
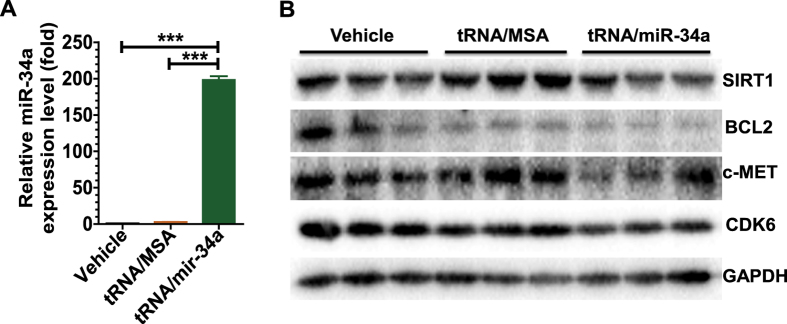
Genetically engineered miR-34a prodrug was processed to mature miR-34a in human osteosarcoma 143B cells (**A**), which consequently reduced the protein expression of miR-34a target genes including SIRT1, BCL2, CDK6 and c-MET (**B**). Cells were harvested at 72 h after treatment with tRNA/mir-34a or control tRNA/MSA, or vehicle. Mature miR-34a levels were quantitated by selective stem-loop reverse transcription RT-qPCR assay, and normalized to U6 levels. Protein was detected by Western blot analyses, and GAPDH was used as a loading control. Values are mean ± SD of triplicate treatments. ***P < 0.001 (one-way ANOVA), as compared with tRNA/MSA or vehicle control treatment.

To understand the molecular mechanisms underlying the antiproliferative effect of bioengineered miR-34a prodrug against OS 143B cells, we quantitated mature miR-34a levels using selective stem-loop reverse transcription RT-qPCR assay and determined the protein levels of several miR-34a targeted oncogenes using Western blot analysis. Our data revealed a 190-fold higher mature miR-34a level in the cells treated with tRNA/mir-34a than tRNA/MSA or vehicle (Fig. 2A), consistent with our recent findings from unbiased RNA sequencing and targeted RT-qPCR studies in lung carcinoma cells^24^. Furthermore, immunoblot analyses showed that 143 cells treated with tRNA/mir-34a had lower protein levels of several miR-34a target genes including SIRT1, BCL2, CDK6, and c-MET (Fig. 2B). These results indicate that the antiproliferative activity of bioengineered miR-34a prodrug (Fig. 1) may be attributable to the processed mature miR-34a and consequently the suppressed target oncogene expression (Fig. 2).

### Bioengineered miR-34a prodrug induces apoptosis in osteosarcoma 143B cells

**Figure 3:**
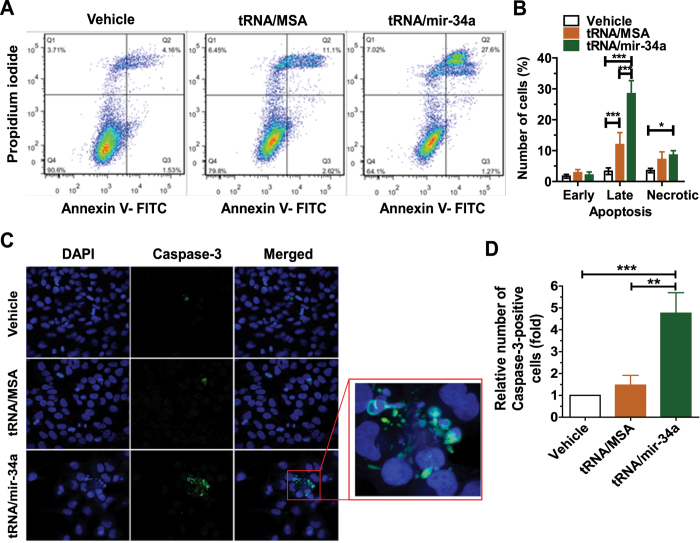
Bioengineered miR-34a prodrug enhanced the apoptosis of osteosarcoma 143B cells. (**A**) Comparison of flow cytometry histograms of Annexin V/propidium iodide stained cells, following the treatments with tRNA/mir-34a, control tRNA/MSA and vehicle. (**B**) Comparison of the percent of apoptotic cells with different treatments. (**C**) Confocal immunofluorescent analyses of Caspase-3 biomarker in 143B cells. (**D**) The number of Caspase-3-positive cells were compared for different treatments. Cells were treated with 4 nM tRNA/mir-34a, tRNA/MSA or the vehicle for 72 h, and apoptosis was assessed by Annexin V/propidium iodide flow cytometric analysis. A separate batch of treated cells were fixed with 10% formalin, stained with Caspase-3 antibody, and counterstained with DAPI. Caspase-3 fluorescence and DAPI-stained nuclei images were acquired with a confocal microscope. Values are mean ± SD of triplicate treatments. *P < 0.05, **P < 0.01, and ***P < 0.001 (two- or one-way ANOVA).

To evaluate whether the inhibition of 143B cell proliferation by miR-34a prodrug involves apoptosis mechanism, we examined apoptotic profiles using Annexin V/propidium iodide flow cytometric analysis (Fig. 3A). While tRNA/mir-34a treatment led to an increase in the number of necrotic cells than the vehicle control, the effect did not differ between BERA tRNA/mir-34 and tRNA/MSA treatments (Fig. 3B). In contrast, tRNA/mir-34a significantly enhanced late apoptosis to much greater degrees, compared with the control tRNA/MSA or vehicle treatment (P < 0.001, two-way ANOVA; Fig. 3B). Thus we further investigated an apoptosis biomarker, Caspase-3, using immunofluorescence assay (Fig. 3C), and quantitatively compared the number of Caspase-3-positive cells between different treatment groups (Fig. 3D). Our data showed that there was a 5-fold increase in Caspase-3-positive cells in tRNA/mir-34a treatment group, as compared with tRNA/MSA or vehicle treatment (P < 0.01, one-way ANOVA; Fig. 3B). Together, the results suggest that miR-34a prodrug largely enhances apoptosis of osteosarcoma 143B cells.

### BERA miR-34a prodrug causes a G2 cell cycle arrest in osteosarcoma 143B cells

**Figure 4:**
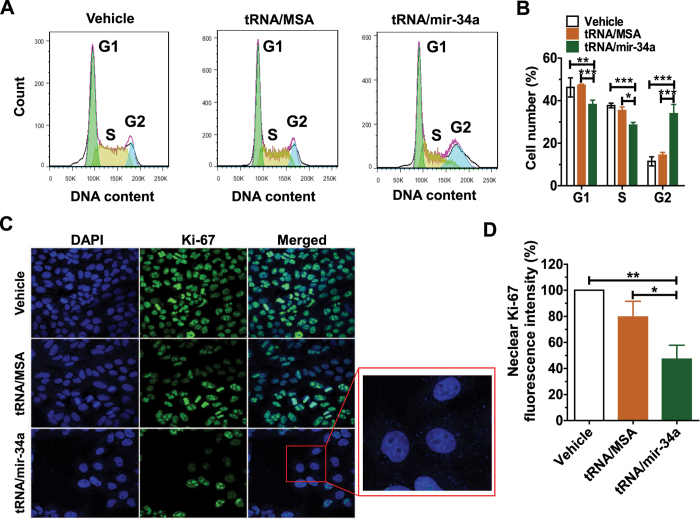
Bioengineered miR-34a prodrug induced a G2 cell cycle arrest in osteosarcoma 143B cells. (**A**) Comparison of flow cytometry histograms of propidium iodide-stained cells treated with vehicle, tRNA/MSA and tRNA/mir-34a. (**B**) Comparison of each cell cycle (G1, S and G2) phase percentage. (**C**) Images of biomarker Ki-67 fluorescence in 143B cells with different treatments. (**D**) Nuclear Ki-67 fluorescence intensities were compared for different treatments, which were quantitated with ImageJ software and normalized to the numbers of cells in corresponding groups. Cells were treated with 4 nM tRNA/mir-34a or tRNA/MSA, or the vehicle for 72 h, stained with propidium iodide, and flow cytometric analyses were conducted to define cell cycle phases. A different batch of treated cells were fixed with 10% formalin, stained with Alexa Fluor dye-labeled Ki-67 antibody, and counterstained with DAPI. Ki-67 fluorescence and DAPI-stained nuclei images were acquired with a confocal microscope. Values are mean ± SD of triplicate treatments. *P < 0.05, **P < 0.01, and ***P < 0.001 (two- or one-way ANOVA), as compared with either tRNA/MSA or vehicle treatment.

To assess whether cell cycle progress is arrested at certain checkpoints by miR-34a prodrug, we investigated the cell cycle profiles with flow cytometric analysis after staining for DNA content (Fig. 4A). Compared with tRNA/MSA or vehicle treatment, tRNA/mir-34a resulted in a 3-fold higher accumulation of 143B cells in G2 phase (P < 0.001, two-way ANOVA; Fig. 4B). The G2 phase arrest was also accompanied with a reduction of cells in G1 and S phases. Likewise, we employed immunofluorescence assay to examine the cell proliferation biomarker Ki-67, a nuclear antigen expressed in all active phases of cell cycle. Consistent with the above MTT study (Fig. 1A), tRNA/mir-34a sharply reduced the number of live 143B cells, as indicated by nuclear staining with DAPI (Fig. 4C). Interestingly, nuclear Ki-67 contents in the cells treated with tRNA/mir-34a were significantly lower than tRNA/MSA or vehicle (P < 0.05, one-way ANOVA; Fig. 4D), demonstrating a compromised proliferation ability. Moreover, the Ki-67 was almost absent in a large portion of tRNA/mir-34a-treated 143B cells (Fig. 4C; highlighted in the red rectangle), which may indicate that these cells were undergoing cell cycle arrest.

### Biological miR-34a prodrug suppresses the invasion ability of osteosarcoma 143B cells

**Figure 5:**
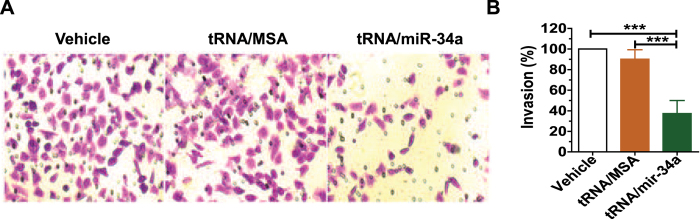
Bioengineered miR-34a prodrug significantly inhibited the invasion ability of osteosarcoma 143B cells. Cell images (A; 200× magnification) were acquired with a Olympus IX2-UCB microscope, and invasion capacities were compared for different treatments (**B**). Cells were treated with 4 nM tRNA/mir-34a or tRNA/MSA, or vehicle for 72 h, subjected to Matrigel invasion assay for 24 h, fixed with 10% formalin, and then stained with 0.1% crystal violet. Values are mean ± SD of six treatments. ***P < 0.001 (one-way ANOVA), as compared with tRNA/MSA or vehicle treatment.

We further investigated the impact of miR-34a prodrug on invasion capability of human OS 143B cells, since cancer cell invasion is a critical process for tumor progression and metastasis. Invasion of 143B cells under different treatments was determined by the Matrigel invasion assay, and invasive ability was indicated by the number of cells presented on the lower side of the insert chamber (Fig. 5). Our data revealed that the invasion ability of 143B cells treated with tRNA/mir-34a was 60–70% lower, compared to cells treated with tRNA/MSA and vehicle (P < 0.001, one-way ANOVA; Fig. 5B). The results suggest that miR-34a prodrug is able to reduce the invasion ability of human OS 143B cells.

### Bioengineered miR-34a prodrug is effective to suppress osteosarcoma growth in an orthotopic xenograft tumor mouse model

**Figure 6:**
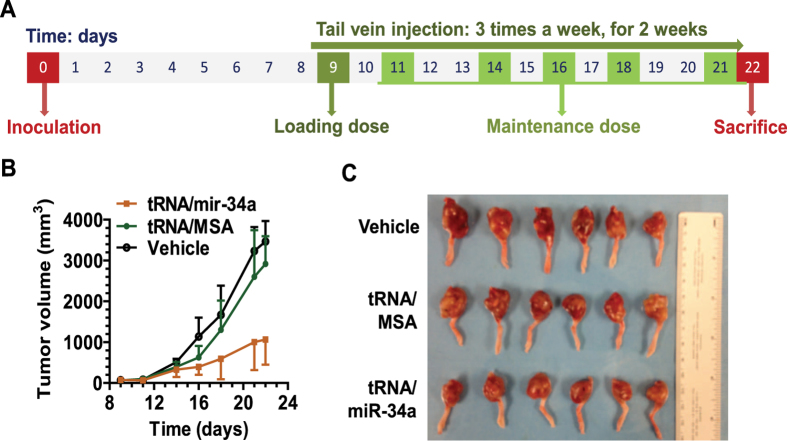
Genetically engineered miR-34a prodrug significantly suppressed tumor growth in an orthotopic osteosarcoma xenograft tumor mouse model. (**A**) Timeline of osteosarcoma cell inoculation and drug treatment. (**B**) Xenograft tumor growth was significantly (P < 0.001, two-way ANOVA) reduced in mice treated with tRNA/miR-34a than the control tRNA/MSA or vehicle. (**C**) Visual comparison of the dissected orthotopic tumor tissues. Values are mean ± SD (N = 6 in each group).

We thus developed an orthotopic OS xenograft tumor mouse model through subperiosteal injection of 143B cells into immunodeficient mice, and evaluated the effectiveness of BERA miR-34a prodrug to control tumor growth *in vivo*. All mice inoculated with 143B cells developed primary tumors, which were readily visualized and reached to 50–110 mm^3^ in 8–10 days. We treated these mice intravenously with *in vivo*-jetPEI-formulated tRNA/mir-34a for 2 weeks (Fig. 6A). Separate groups of animals bearing 143B xenograft tumors were treated with the same doses of *in vivo*-jetPEI-formulated tRNA/MSA or vehicle as controls. Compared with the vehicle or the same doses of tRNA/MSA, tRNA/mir-34a treatment showed a remarkable suppression of the outgrowth of viable tumors (P < 0.001, two-way ANOVA; Fig. 6B). Dissected orthotopic xenograft tumors from tRNA/mir-34a-treated mice were indeed much smaller (Fig. 6C) than tRNA/MSA and vehicle treatment groups, which were further confirmed by histological examination (data not shown). These results indicate that intravenous administration of biological miR-34a prodrug is effective to control orthotopic OS xenograft tumor progression *in vivo*.

### Therapeutic doses of miR-34a prodrug are well tolerated in xenograft tumor mice

**Figure 7:**
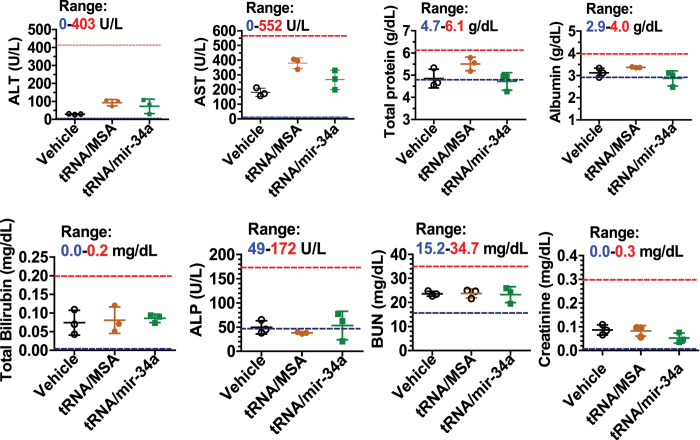
Administration of therapeutic doses of bioengineered miR-34a prodrug had no significant effects on mouse blood chemistry profiles including alanine transaminase (ALT), aspartate transaminase (AST), total protein, albumin, total bilirubin, alkaline phosphatase (ALP), blood urea nitrogen (BUN), and creatinine levels, as compared to vehicle or tRNA/MSA treatment. The ranges of individual markers (derived from BALB/c mice; Comparative Pathology Laboratory at UC-Davis) were labeled as a reference. Values are mean ± SD (N = 3 in each group).

In addition, therapeutic doses of miR-34a prodrug had no significant effect on mouse body weights, compared with either vehicle or tRNA/MSA treatments (data not shown). Mouse blood chemistry profiles including alanine aminotransferase (ALT), aspartate aminotransferase (AST), total protein, albumin, total bilirubin, alkaline phosphatase (ALP), blood urea nitrogen (BUN), and creatinine were further determined and compared between different treatment groups. The data showed that none of these blood biomarkers was significantly altered by biological tRNA/mir-34a, as compared with vehicle or tRNA/MSA treatments (Fig. 7), suggesting the absence of liver or kidney toxicity. These results indicate that therapeutic doses of miR-34a prodrug are well tolerated in the orthotopic OS xenograft tumor mouse models during the treatment.

## Discussion

Evaluation of miRNA based therapies in animal models and human subjects relies on the access to large quantities of miRNA agents. The novel BERA miR-34a prodrug examined in current study is a natural RNA agent produced and folded within the cells^24^,^25^, which are distinguished from synthetic miR-34a mimics and precursors^16^,^19^,^29^,^30^,^35^ consisting of extensive chemical modifications on the ribose and phosphate bone that may alter the tertiary structure, physicochemical properties, biological/pharmacological activity, and/or safety profiles^31^. In addition, bioengineered miR-34a prodrug is literally large molecule RNA agent, in contrast to viral or non-viral vector-based miR-34a expression plasmids/systems^36^ that are rather DNA materials and may complicate these RNA-based processes. Therefore, bioengineered pre-miRNAs such as miR-34a prodrug investigated for OS in current study may represent a new class of large molecules for therapy.

Investigation of new therapeutics also requires the use of more clinical relevant models. Many human OS cell lines have been critically assessed for relevance to the characteristics of primary OS in patients^32^,^37^. The 143B cells carrying a *p53* mutant and showing high-level expression of p53 were consistently identified to be highly tumorigenic and metastatic, and thus it is widely used to study OS cancer biology and examine new therapies^32^,^33^,^34^,^38^,^39^,^40^,^41^. By contrast, the MG-63 cells were found to be tumorigenic in mice by some studies^15^,^42^,^43^ but unable to produce xenograft tumors by other studies^32^, whose morphology is actually more fibroblastic and consists of wild-type *p53* and lower level of p53 expression. Our results showed that 143B cells were more sensitive to miR-34a prodrug than MG-63 cells *in vitro*, which may be related to the involvement of miR-34a in the p53 pathways^17^,^23^,^44^. Indeed all mice inoculated with 143B cells in our study developed xenograft tumors for drug efficacy and safety tests *in vivo*. Nevertheless, none of the mice showed any lung metastases, which may be due to an early termination (22 days after inoculation) and injection of less cells (1.5 × 10^6^ of 143 cells per mouse) than previous studies (1 month after inoculation and 2.0 × 10^6^ of 143 cells per mouse, respectively)^32^.

Using the orthotopic 143B xenograft OS tumor mouse model and genetically engineered miR-34a prodrug, this study revealed that miR-34a replacement therapy was indeed effective to reduce the growth of OS xenograft tumors, supporting the concept to develop novel biological miRNA therapeutics^31^. Current study is different from our recent study in which mice bearing subcutaneous xenograft lung tumors were treated intratumorally with recombinant miR-34a^24^. In this regard, the present study on systemic administration of miR-34a prodrug to orthotopic xenograft tumor mouse is more relevant to clinical applications. Additionally, therapeutic doses of biological miR-34a prodrug were well tolerated in these tumor mice, as manifested by the absence of change in blood chemistry profiles and signs of any stress (e.g., hunched posture and labored movement). This finding is also different from our recent study on the safety profiles of an acute, high dose of bioengineered miR-34a prodrug in immunocompetent mice^24^.

Consistent with our recent observations in human lung adenocarcinoma cells^24^, the present study also showed that tRNA-carried miR-34a prodrug was selectively processed to high levels of mature miR-34a in OS cells. The mechanistic actions of miR-34a were supported by the selective suppression of protein expression levels of a number of well-defined miR-34a target genes including SIRT1, BCL2, CDK6, and c-MET that are critical for cancer cellular processes, such as cell cycle, apoptosis and invasion^17^,^18^,^19^,^20^. Therefore, the reduction of miR-34a target genes also provides a molecular explanation for the effects of miR-34a prodrug in inhibiting OS cell proliferation, enhancing apoptosis, inducing cell cycle arrest, and suppressing invasion ability in cells *in vitro*, which together offers a good explanation for the effectiveness of miR-34a in suppressing OS xenograft tumor growth in mice *in vivo*.

It is noteworthy that the tRNA scaffold itself, at higher concentrations, was able to inhibit the proliferation of OS cells (current study) as well as lung and liver cancer cells^24^, whereas to a much lower degree than the tRNA/mir-34a. The genomically-derived tRNA species are eventually processed to tRNA fragments (tRFs) within mammalian cells, which may be induced under hypoxic stress^45^,^46^,^47^,^48^. Some tRFs were shown to inhibit cancer cell proliferation, angiogenesis or metastasis^45^,^48^,^49^. The antiproliferative activity of this methionyl tRNA (tRNA/MSA; Fig. 1) is attributable to the induction of apoptosis (Fig. 3), which is also processed to various tRFs in human carcinoma cells^24^,^25^. A very recent report showed that several tRF species were effective to suppress the tumorigenesis of breast cancer cells via binding to oncogenic RNA-binding protein, YBX-1, to displace pro-oncogenic transcripts^48^. However, the present study showed that lower doses of intravenously administered tRNA/MSA did not affect orthotopic OS xenograft tumor progression (Fig. 6), consistent with recent observations from subcutaneous lung and liver xenograft tumor mouse models^24^.

In summary, the present study demonstrated that systemic administration of therapeutic doses of highly purified, genetically engineered miR-34a prodrug was effective to suppress tumor growth and well tolerated in the orthotopic osteosarcoma xenograft tumor mouse models. Mechanistically, BERA miR-34a prodrug was processed to mature miR-34a in human osteosarcoma cells and subsequently reduced the protein expression of many miR-34a target genes, enhanced apoptosis, induced cell cycle arrest, and inhibited cell invasion capability. These findings support the miR-34a replacement therapy strategy for the treatment of osteosarcoma and most importantly, the development of bioengineered miRNA agents as novel large molecule therapeutics.

## Methods

### Human cell lines

The human OS cell lines 143B (CRL-8303) and MG-63 (CRL-1543) were purchased from American Type Culture Collection (Manassas, VA). Both 143B and MG-63 cells were cultured in RPMI 1640 medium (GE Healthcare Bio-Sciences, Pittsburgh, PA) supplemented with 10% fetal bovine serum (GBICO BRL, Rockville, MD), at 37 °C in a humidified atmosphere containing 5% CO_2_. Cells in the logarithmic growth phase were used for experiments.

### Bioengineering miR-34a prodrug

Expression and purification of biological miR-34a prodrug (tRNA/mir-34a or sephadex aptamer tagged methionyl tRNA fusion pre-miR-34a) and control tRNA scaffold (tRNA/MSA or sephadex aptamer tagged methionyl tRNA) were conducted as we described very recently^24^,^25^,^26^. The purity was verified by denaturing urea (8 M) polyacrylamide (8%) gel electrophoresis (PAGE) and high performance liquid chromatography (HPLC) analyses^24^, and highly purified (over 98% by HPLC) recombinant noncoding RNAs (ncRNAs) were used in this study.

### MTT assay

The effects of bioengineered miR-34a prodrug on the proliferation of 143B and MG-63 OS cells were determined using MTT assay, as described^24^,^25^,^26^. Briefly, cells were seeded in 96-well plates (Corning, NY) at 5,000 cells per well in 200 μl medium. 24 h later, cells were treated with various concentrations (0, 0.3, 1.0, 3.0, 5.0, 10, 30, and 100 nM) of tRNA/mir-34a or tRNA/MSA using Lipofectamine 2000 (Thermo Fisher Scientific Inc.) for 72 h. Cell viability was then determined using a MTT Cell Proliferation Assay kit (Thermo Fisher Scientific Inc.). Cells were treated in triplicate and assayed separately. Data were fit into a normalized inhibitory dose-response model with variable slope (Y = Bottom + (100-Bottom)/(1 + 10^((LogEC50-X)*HillSlope); GraphPad Prism, San Diego, CA) for the estimation of EC50, Hill slope, and Bottom values.

### RNA isolation and reverse transcription quantitative real-time PCR (RT-qPCR)

OS 143B cells were seeded in six-well plates (0.1 × 10^6^ cells per well). 24 h later, cells were transfected with 10 nM tRNA/mir-34a or tRNA/MSA, or vehicle. Cells were treated for 72 h, and total RNAs were isolated using the Direct-zol RNA MiniPrep kit (Zymo Research, Irvine, CA). Reverse transcription was performed using NxGen M-MuLV reverse transcriptase (Lucigen, Middleton, WI), and a stem-loop primer 5′-GTCGTATCCAGTGCAGGGTCCGAGGTATTCGCACTGGATACGACACAACC-3′ (IDT, San Diego, CA) for miR-34a, and iScript reverse-transcription Supermix (Bio-Rad) for U6. RT-qPCR was performed using quantitative RT-PCR Master mix (New England Biolabs) on a CFX96 Touch real-time PCR system (Bio-Rad), as described previously^24^,^25^. Cells were treated in triplicate and assayed separately. Relative miR-34a levels were calculated by using the comparative threshold cycle (Ct) method with the formula 2^−∆∆Ct^.

### Protein isolation and Western blot analysis

Cells were seeded in six-well plates and treated with 4 nM tRNA/mir-34a, tRNA/MSA or vehicle, as described above. 72 h post-transfection, cells were harvested with trypsin and washed twice with cold PBS. Cell lysates were prepared using RIPA buffer (Rockland Immunochemical Inc., Limerick, PA) supplemented with the complete protease inhibitor cocktail (Roche, Nutley, NJ). Protein concentrations were quantitated with a BCA Protein Assay Kit (Thermo Fisher Scientific Inc.). Whole cell proteins (40 μg/lane) were separated on a 10% SDS-PAGE gel and electro-transferred onto PVDF membranes using Trans-Blot Turbo Transfer System (Bio-Rad). Membranes were incubated with anti-SIRT1 (1:500 dilution; H-300; Santa Cruz Biotech Inc., Texas, TX), anti-c-MET (1:200; C-28; Santa Cruz), anti-BCL2 (1:200; N-19; Santa Cruz), anti-CDK6 (1:1000; C-21; Santa Cruz), or anti-GAPDH (1:2,000; FL-335; Santa Cruz) rabbit polyclonal antibody. The membranes were then blotted with a peroxidase goat anti-rabbit IgG (Jackson ImmunoResearch Inc., West Grove, PA), incubated with Clarity Western ECL substrates (Bio-Rad), and visualized with the ChemiDoc MP Imaging System (Bio-Rad).

### Flow cytometry analyses of cell cycle and apoptosis

Cells were seeded in six-well plates and treated with 4 nM tRNA/mir-34a or tRNA/MSA, or vehicle for 72 h. To examine the effects on cell cycle, DNA of the cells was stained with PI/RNase Staining Buffer (BD Biosciences, San Jose, CA). To define the impact on apoptosis, cells were incubated with Annexin V-FITC conjugate and propidium iodide solution (Trevigen Inc., Gaithersburg, MD). Samples were analyzed on a FACScan flow cytometer (BD Biosciences, San Jose, CA), and all data were processed by Flowjo (Ashland, OR). Cells were treated in triplicate and assayed separately.

### Cell invasion assay

Cells were treated with 4 nM tRNA/mir-34a or tRNA/MSA, or vehicle. After 72 h, 3 × 10^4^ cells were subjected to invasion assay using the Corning BioCoat Matrigel Invasion Chamber with 8.0 μm PET membrane (Corning, NY) for 24 h. The upper inserts with matrigel coating were then fixed with 10% formalin, stained with 0.1% crystal violet, and photographed under an Olympus IX2-UCB microscope (200× magnification). Cells were treated in triplicate and assayed separately. Five fields per insert were photographed. The number of cells was counted using Adobe Photoshop’s count tool, and compared between different treatments as we described recently^50^.

### Immunofluorescence study on apoptosis and proliferation biomarkers

Cells were seeded onto gelatin coated coverslips (Neuvitro Corporation, Vancouver, WA), which were inserted in 6-well plates. Cells were then treated with 4 nM tRNA/mir-34a or tRNA/MSA, or vehicle or 72 h. The coverslips were fixed with 10% formalin, permeabilized with 0.5% tween-20 (for Caspase-3, located in the cytoplasm) or Triton-X100 (for Ki-67, located in the nucleus), and blocked with 1% BSA. After that, coverslips were incubated overnight with a primary monoclonal antibody (anti-Caspase-3, #9579S; anti-Ki-67, 9129S; Cell signaling Technology, Beverly, MA), and then with a secondary antibody (anti-rabbit IgG Alexa Fluor® 488 Conjugate, #4412; Cell signaling Technology). Nucleus was conterstained with DAPI (#8961, Cell signaling Technology). Images were taken with a Zeiss Axio Observer.z1 Microscope coupled to a Zeiss LSM 710 Scanning Device (Zeiss, Oberkochen, Germany). Cells were treated in triplicate and assayed separately. Caspase-3-positive cells were counted directly under the confocal microscope. The intensity of Ki-67 fluorescence was quantified using the ImageJ software, normalized to the number of cells/nuclei, and then compared between different treatments.

### Orthotopic osteosarcoma xenograft tumor mouse model

Animal procedures were approved by the Institutional Animal Care and Use Committee (IACUC) at UC-Davis, and were conducted in accordance with the Guide for the Care and Use of Laboratory Animals issued by the National Institutes of Health. Immunodeficient athymic nude mice (5- to 6-week old males, J:NU strain) were purchased from The Jackson Laboratory (Bar Harbor, ME). Orthotopic OS xenograft tumors were generated using a subperiosteal injection method^22^,^38^. Briefly, 143B cells (4–6 passages) were harvested and resuspended in PBS to a final concentration of 3 × 10^7^ cells/ml. 50 μl of the cell suspension (1.5 × 10^6^ cells) were mixed with 50 μl of matrigel matrix (BD Biosciences). Animals were anesthetized i.p. with ketamine (80 mg/kg) and xylazine (7 mg/kg). 100 μl of the above prepared cell suspension were injected subperiosteally onto the right proximal lateral tibia using a 25 gauge needle (BD Biosciences). Gentle pressure was applied onto the injection point with index finger for 10 seconds to prevent the mix from oozing out. The injection sites were disinfected afterward.

### Therapeutic drug efficacy and safety studies

Tumor size was measured with a caliper, and tumor volume was calculated using the equation: tumor volume (mm^3^) = (length + width) (mm) × length (mm) × width (mm) × 0.2618)^39^. On day 9 post-inoculation, 18 mice with similar body weights and tumor sizes (tumor volumes = 69.3 ± 35.4 mm^3^) were randomly divided into 3 groups. Bioengineered tRNA/mir-34a was formulated with *in vivo*-jetPEI (Polyplus-transfection Inc., New York, NY), and administered i.v. into the mice via tail vein (loading dose 50 μg/mouse on day 9; maintenance dose 25 μg/mouse/day on days 11, 14, 16, 18, and 21). Control mice were treated with the same doses of tRNA/MSA formulated with *in vivo*-jetPEI or just the vehicle. On day 22 post inoculation, all mice were sacrificed and xenograft tumors were dissected and visually compared. Tumors were fixed in 10% formalin and subjected to hematoxylin and eosin staining histological examination in the Clinical Immunohistochemistry Laboratory at Roswell Park Cancer Institute. In addition, blood sample was collected from each mouse, and serum was isolated using a serum separator (BD Biosciences). Mouse blood chemistry profiles were determined in the Comparative Pathology Laboratory at UC-Davis.

### Statistical analysis

Values were expressed as mean ± SD. Depending upon the groups and variances, data were analyzed with one-way or two-way ANOVA with Bonferroni post-tests (GraphPad Prism). Difference was considered as significant when P-value was less than 0.05 (P < 0.05).

## Additional Information

**How to cite this article**: Zhao, Y. *et al*. Genetically engineered pre-microRNA-34a prodrug suppresses orthotopic osteosarcoma xenograft tumor growth via the induction of apoptosis and cell cycle arrest. *Sci. Rep.* **6**, 26611; doi: 10.1038/srep26611 (2016).
